# Down-regulation of HDAC3 inhibits growth of cholangiocarcinoma by inducing apoptosis

**DOI:** 10.18632/oncotarget.19660

**Published:** 2017-07-28

**Authors:** Mingming Zhang, Yuyao Yin, Robert G. Dorfman, Tianhui Zou, Yida Pan, Yang Li, Yuming Wang, Qian Zhou, Lixing Zhou, Bo Kong, Helmut Friess, Jun Zhang, Shimin Zhao, Lei Wang, Xiaoping Zou

**Affiliations:** ^1^ Department of Gastroenterology, Nanjing Drum Tower Hospital, The Affiliated Hospital of Nanjing University Medical School, Nanjing University, Nanjing, China; ^2^ Key Laboratory of Reproduction Regulation of NPFPC (SIPPR, IRD), Shanghai, China; ^3^ Department of Digestive Diseases of Huashan Hospital, Shanghai, China; ^4^ School of Life Sciences, Fudan University, Shanghai, China; ^5^ Northwestern University Feinberg School of Medicine, Chicago, IL, USA; ^6^ Department of Gastroenterology, Renji Hospital, Shanghai Jiaotong University, Shanghai, China; ^7^ Department of Surgery, Technical University of Munich (TUM), Munich, Germany

**Keywords:** apoptosis, cholangiocarcinoma, HDAC3, prognosis

## Abstract

Class I histone deacetylases (HDACs) inhibit expression of tumor suppressor genes by removing acetyl groups from histone lysine residues, thereby increasing cancer cell survival and proliferation. We evaluated the expression of class I HDACs in cholangiocarcinoma (CCA). HDAC3 expression was specifically increased in CCA tissues and correlated with reduced patient survival. HDAC3 overexpression inhibited apoptosis and promoted CCA cell proliferation. Conversely, HDAC3 knockdown or pharmacological inhibition decreased CCA cell growth and increased caspase-dependent apoptosis. Inhibition of class I HDACs blocked HDAC3-catalyzed deacetylation and increased expression of downstream pro-apoptotic targets *in vitro* and *in vivo*. These results demonstrate for the first time that down-regulation of HDAC3 induces apoptosis in human CCA cells, indicating that inhibiting HDAC3 may be an effective therapeutic strategy for treating CCA .

## INTRODUCTION

Cholangiocarcinoma (CCA) is a highly malignant adenocarcinoma of mostly unknown etiology, with increasing mortality in many countries [1–3]. Most patients are diagnosed at late stages and are not eligible for surgical treatment. As a result, 5-year survival rates of CCA have remained at 10% for the past three decades [4]. Even though chemotherapy has been effective in some CCA patients, many patients are not responsive to conventional chemotherapies, emphasizing the urgent need for novel therapeutic strategies for CCA [1–4].

Epigenetic changes, including histone modifications, play an important role in malignant adenocarcinomas [5]. Histone deacetylases (HDACs) catalyze the removal of acetyl groups from lysine tails. Based on their DNA homology, HDACs are divided into class I, II and IV. Class I HDACs (1, 2, 3, and 8) are expressed in many types of cancer; they play an important role in tumorigenesis [6–11]. Since class I HDACs inhibit specific tumor suppressor genes, resulting in an aberrant epigenetic status of cancer cells compared to healthy cells, they may serve as candidate anti-cancer targets [12, 13]. Increased expression and activation of HDAC3 play a critical role in epigenetic alterations associated with different types of malignancies [14, 15]. However, the role of HDAC3 in CCA has not yet been elucidated.

HDACs inhibitors induce cell cycle arrest and apoptosis in a broad spectrum of cancer cells [16–19]. Vorinostat (suberoylanilide hydroxamic acid, SAHA) and romidepsin have been approved by the U.S. Food and Drug Administration (FDA) for the treatment of cutaneous T-cell lymphoma [20]. Considering their high anticancer activity in hematological malignancies, it is anticipated that they will find applications for the treatment of solid cancers as well. Novel class I HDACs inhibitors (4SC202, BG45, SBHA) have exhibited an anti-cancer activity both *in vitro* and *in vivo,* with marginal toxicity [5, 21, 22]. However, the effects of class I HDACs inhibitors in CCA have not yet been studied.

Here we show that high protein levels of HDAC3 in CCA tissues are associated with poor survival in patients with CCA. Increased expression of HDAC3 induces proliferation and inhibits apoptosis in CCA cells. Down-regulation of HDAC3 induces apoptosis of CCA cells, resulting in a reduced CCA growth. Together, our findings indicate that HDAC3 induces CCA progression by promoting cell proliferation, and suggest that it may serve as a potential target for therapeutic intervention in the treatment of CCA.

## RESULTS

### HDAC3 expression is increased in CCA tissues, and associated with reduced patient survival

**Figure 1:**
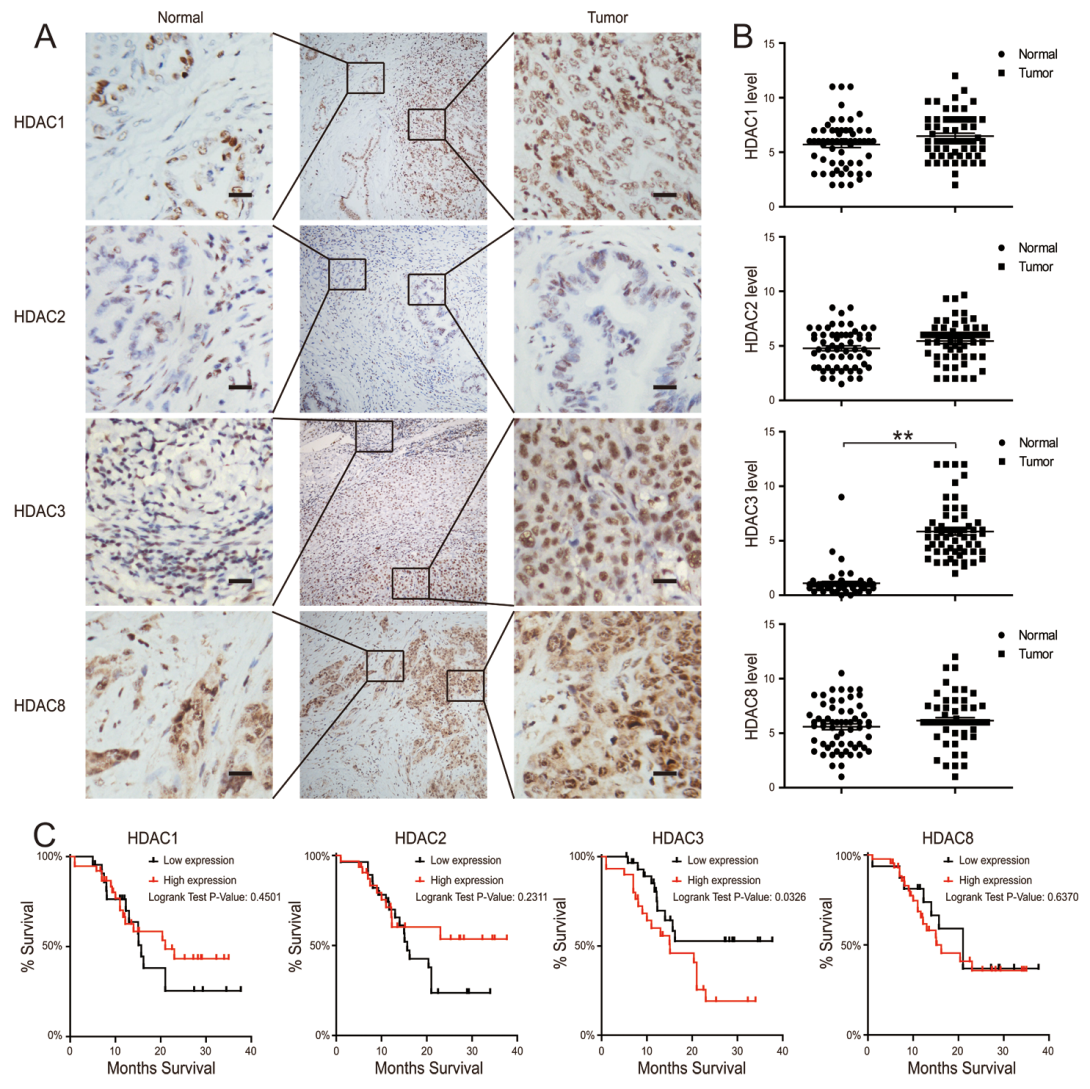
HDAC3 expression is increased in CCA tissues **(A and B)** HDAC1, 2, 3 and 8 protein levels in tumor and adjacent normal tissues from 60 CCA patients were analyzed by IHC (left) and quantified (right). The magnification is x200. Scale bars, 100 μm. **(C)** 3-year survival was evaluated for CCA patients with HDAC1, 2, 3 and 8 protein expression. Data represent the mean ± SEM, n≥3. *p<0.05, **p<0.01.

We used CCA tissues from the Biobank of Nanjing Drum Tower Hospital, which contains clinically annotated data from 60 CCA samples; the clinical characteristics of the study participants are summarized in Table 1 . Using immunohistochemistry (IHC), we found that there was no difference in the expression of HDAC1, HDAC2, or HDAC8 isoenzymes between CCA tissues and their adjacent tissues (Figure 1A & 1B). Based on the illustrated frequency distribution, there was no difference between the high and low HDAC3 groups with respect to age, sex, histological differentiation grade, tumor size, nodal metastasis, or pathological stage (Table 1). However, when we assessed the expression of HDAC3, we found that it was significantly increased in CCA tissues compared to adjacent tissues (Figure 1A & 1B). Importantly, the increased HDAC3 expression was associated with a reduced patient survival, whereas other class I HDACs had no correlation with survival (Figure 1C). These findings indicate that an increased HDAC3 expression in CCA tissues is an independent predictor of a poor prognosis in CCA patients.

**Table 1:** Clinical characteristics and HDAC3 levels in patients with cholangiocarcinoma

	HDAC3 expression, n (%)				
	n	Low (%)	High (%)	X^2^	P value
Gender				2.986	0.117
Male	33	22 (66.7)	11 (33.3)		
Female	27	12 (44.4)	15 (55.6)		
Age				1.086	0.435
≤60	26	15 (57.7)	11 (42.3)		
>60	34	15 (44.1)	19 (55.9)		
Location				1.178	0.555
Intrahepatic	24	12 (50.0)	12 (50.0)		
Hepatic portal	25	11 (44.0)	14 (56.0)		
Extrahepatic	11	7 (63.6)	4 (36.4)		
Size (mm)				6.733	0.016
≥40	23	7 (30.4)	16 (69.6)		
<40	37	24 (64.9)	13 (35.1)		
Differentiation				0.315	0.854
Well	7	4 (57.1)	3 (42.9)		
Medium	32	15 (46.9)	17 (53.1)		
Poor	21	11(52.4)	10 (47.6)		
T Stage				1.491	0.36
T1∼T2	46	25(54.3)	21 (45.7)		
T3∼T4	14	5 (35.7)	9 (64.3)		
Lymph node metastasis				2.052	0.252
Negative	43	24 (55.8)	19 (44.2)		
Positive	17	6 (35.3)	11 (64.7)		
Distant metastasis				0	1
Negative	56	28 (50.0)	28 (50.0)		
Positive	4	2 (50.0)	2 (50.0)		
Venous invasion				0	1
Negative	36	18 (50.0)	18 (50.0)		
Positive	24	12 (50.0)	12 (50.0)		
Nerve invasion				0.3	0.785
Negative	20	11 (55.0)	9 (45.0)		
Positive	40	19 (47.5)	21 (52.5)		

### HDAC3 promotes growth of CCA cells

**Figure 2:**
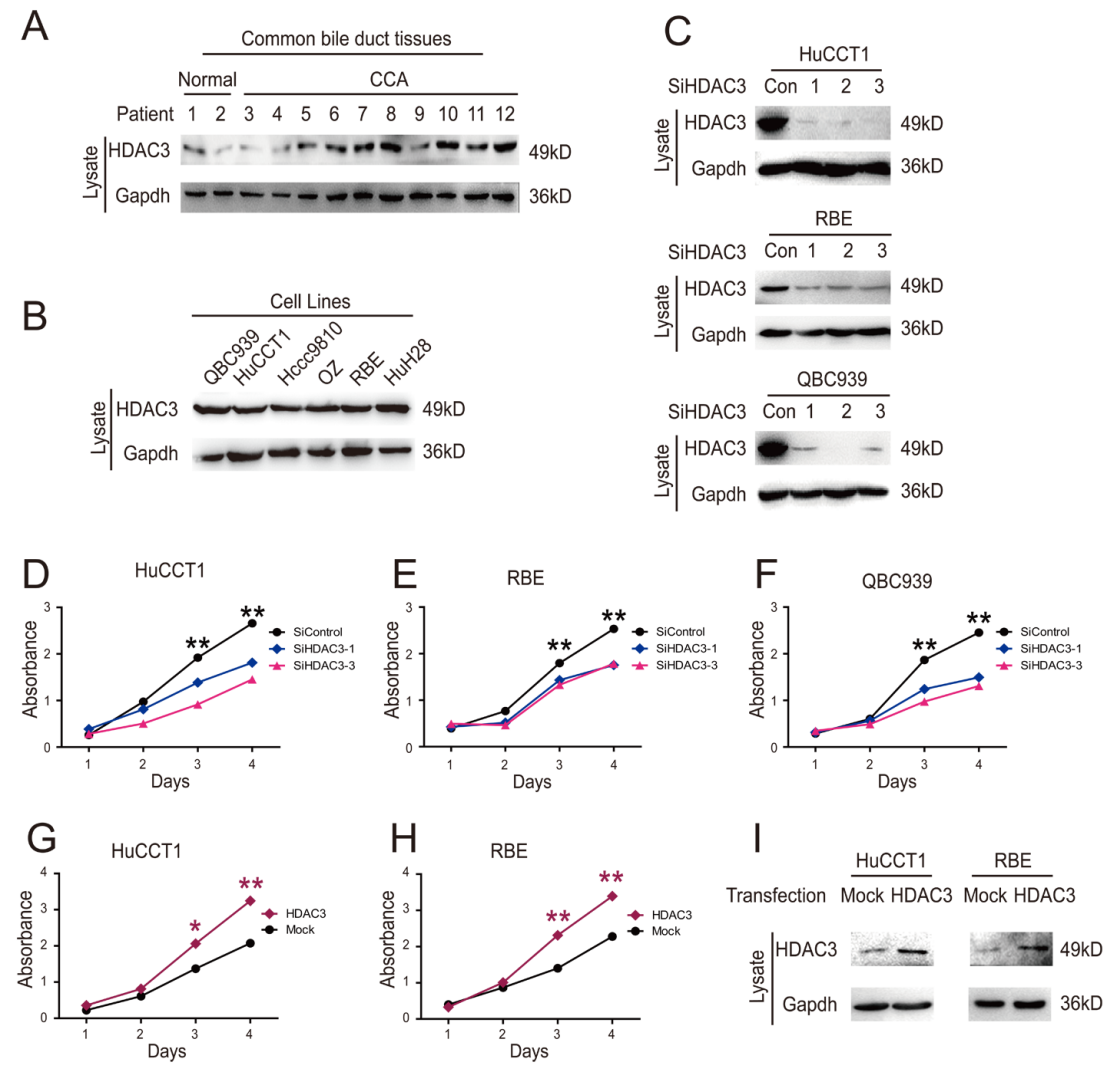
HDAC3 promotes growth of CCA cells **(A)** HDAC 3 protein levels in common bile duct tissues from 12 donors were measured by western blotting. **(B)** HDAC3 protein levels were detected by western blot. **(C)** The knockdown efficiency of HDAC3 siRNA was detected by western blot. **(D, E and F)** CCK8 assay was employed to analyze HuCCT1, RBE and QBC939 cell proliferation following siRNA transfection. **(G and H)** HuCCT1 and RBE cell proliferation was analyzed via CCK8 assay following transfection with HDAC3 overexpression vector. **(I)** Transfection efficiency was confirmed by western blotting. Data represent the mean ± SEM, n≥3. *p<0.05, **p<0.01.

High levels of HDAC3 expression and activity play a critical role in cell epigenetic alterations associated with malignancies [14, 15]. However, the role of HDAC3 in CCA has not been elucidated. We assessed the expression of HDAC3 in different CCA cell lines and fresh tissues by western blotting. HDAC3 protein levels were increased in CCA tissues compared to normal tissues (Figure 2A), and all six CCA cell lines expressed high protein levels of HDAC3 (Figure 2B).

Transfection of CCA cells HuCCT1 and RBE with HDAC3 specific siRNA significantly decreased the HDAC3 expression (Figure 2C), resulting in decreased cell proliferation (Figure 2D, 2E & 2F). In contrast, overexpression of HDAC3 increased CCA cell proliferation (Figure 2G, 2H & 2I). Collectively, these results indicate that HDAC3 increases proliferation of CCA cells.

### HDAC3 inhibition reduces CCA cell viability

**Figure 3:**
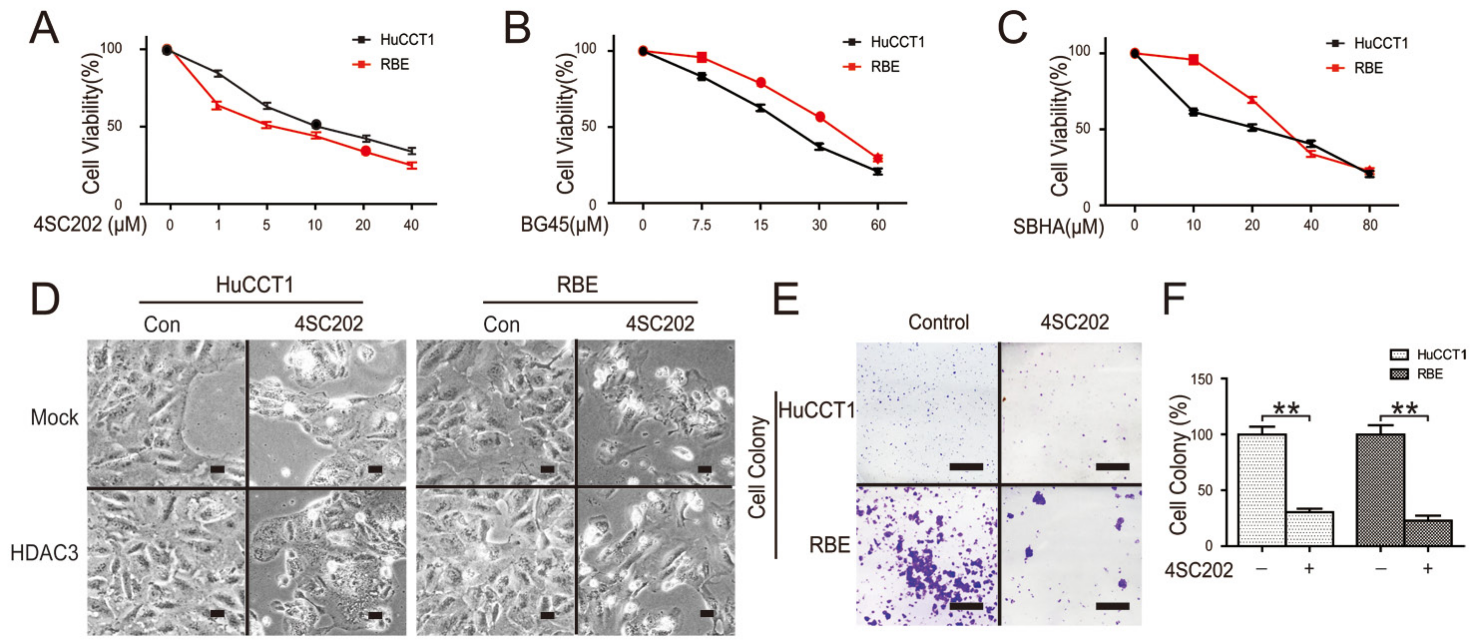
HDAC3 inhibition reduces CCA cell viability **(A, B and C)** Cells were treated with class I HDACs inhibitors (4SC202, BG45, SBHA) and the viability was quantified via CCK-8 assay. **(D)** Cells were transfected with HDAC3 overexpression vector, treated with 4SC202, and morphological changes were analyzed by microscopy using magnification x200 (scale bars, 100 μm). **(E and F)** Cells were treated with 4SC202, colonies were stained with crystal violet (left) and quantified (right). Scale bars, 1 cm. Data represent the mean ± SEM, n≥3. *p<0.05, **p<0.01.

Novel class I HDACs inhibitors (4SC202, BG45, SBHA) have a promising anti-cancer activity with marginal toxicity [5, 21, 22]. 4SC-202 is an orally available potent HDAC class I inhibitor (NO HDAC8) [5]. BG45 is a class I HDAC inhibitor, for which the 50% inhibitory concentrations (IC50) for HDAC1, HDAC2, and HDAC3 are 2.0 μM, 2.2 μM, and 289 nM, respectively[22]. SBHA (suberohydroxamic acid) is a class I HDACs inhibitor selective for HDAC1 and HDAC3; its IC50 for HDAC1 and HDAC3 are 0.25 and 0.3 μM, respectively [21]. Cell viability assay demonstrated that the IC50 of these class I HDAC inhibitors at 48 h was approximately 10 μM in HuCCT1and RBE cells, suggesting that HDAC3 blocking played a key role in inhibiting cell proliferation (Figure 3A-3C). Moreover, overexpression of HDAC3 significantly increased CCA cell growth (Figure 3D). Consistent with CCK8 assays, morphological death was increased, and colony formation in CCA cells was significantly decreased following 4SC202 treatment (Figure 3D-3F). Collectively, these results demonstrate that HDAC3 inhibition reduces CCA cell viability.

### HDAC3 inhibition induces CCA cell apoptosis *in vitro*

**Figure 4:**
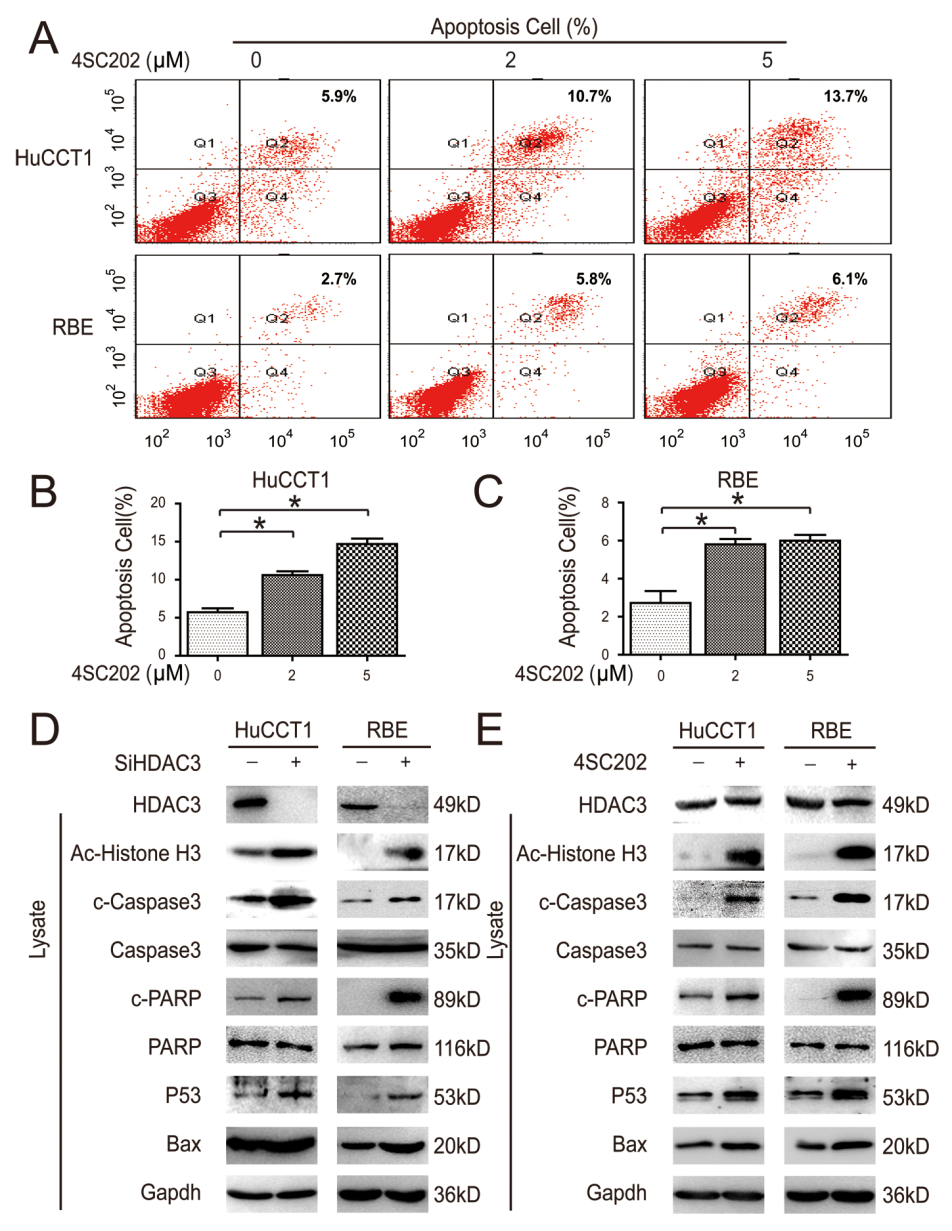
HDAC3 inhibition induces CCA cell apoptosis *in vitro* **(A)** Cells were treated with 4SC202, and cell apoptosis was analyzed via flow cytometry. **(B and C)** Cell apoptosis was quantified. **(D and E)** Cells were collected and subjected to western blot after 4SC202 treatment (left) and siRNA transfection (right).

Flow cytometry was employed to investigate the mechanism of 4SC202 inhibition of CCA cell proliferation. 4SC202 increased apoptosis in two different CCA cell types (Figure 4A-4C). Next, we investigated the effect of HDAC3 inhibition on the downstream targets, including acetylated α-Histone3, P53 and Bax [23]. HDAC3 knockdown increased caspase substrate (polyADP ribose polymerase (PARP) and caspase 3) cleavage and P53 expression; this effect was mimicked by 4SC202 treatment (Figure 4D & 4E). These results indicate that the apoptosis of CCA cells is primarily induced by HDAC3 inhibition, and that HDAC3 may be the dominant target of 4SC202.

### HDAC3 is the main target in 4SC202-induced apoptosis in CCA cells

**Figure 5:**
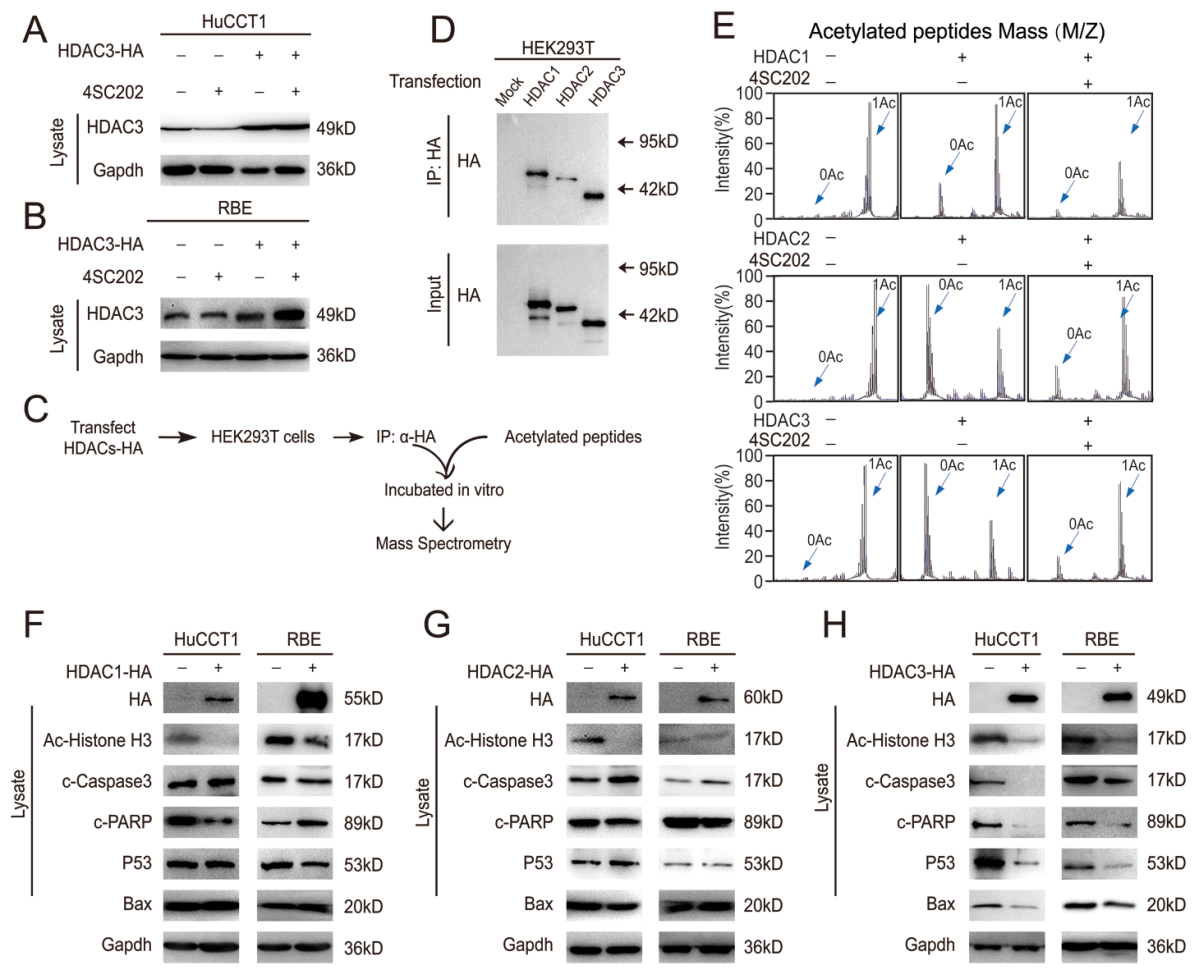
HDAC3 is the main target in CCA cell apoptosis **(A and B)** HDAC3-overexpressing cells were treated with 4SC202 and subjected to western blot. **(C)** Schematic diagram of the *in vitro* deacetylation assay with HDAC3 (top). The immunoprecipitated protein corresponding to HDACs-HA was subjected to western blot (bottom). **(D)** The immunoprecipitated protein corresponding to HDACs-HA was subjected to western blot. **(E)** The HDACs protein was incubated with acetylated peptides with or without 4SC202, and the rate of deacetylation was determined using Mass Spectrometry (MS). **(F)** HDAC1-overexpressing cells and their counterparts were subjected to western blot. **(G)** HDAC2-overexpressing cells and their counterparts were subjected to western blot. **(H)** HDAC3-overexpressing cells and their counterparts were subjected to western blot. Data represent the Mean ± SEM, n≥3. *p<0.05, **p<0.01, NS not significant.

To determine if the pro-apoptotic effect of 4SC202 in CCA cells was due to HDAC3 inhibition, CCA cells were treated with 4SC202 following transfection with HA-tagged HDAC3 vector. HDAC3 protein levels were unchanged by 4SC202 treatment (Figure 5A & 5B), suggesting that 4SC202 inhibits the activity of HDAC3 directly. Due to the high level of homology between the class I HDACs (HDAC 2 shares 52% identity with HDAC3 [24–26]), 4SC202 possibly has a weak inhibitory effect also on HDAC1 and 2 [27]. We therefore sought to determine whether HDAC3 was responsible for the 4SC202-induced apoptosis. To elucidate the direct target of 4SC202, we used an *in vitro* deacetylation system (Figure 5C). 4SC202 treatment inhibited HDAC3 deacetylation activity, but only had a marginal inhibitory effect on HDAC1 and 2 (Figure 5E). We examined the effects of HDACs 1, 2 and 3 on apoptosis related targets and found that only HDAC3 could rescue apoptosis in CCA cell lines (Figure 5F-5H). These results demonstrate that HDAC3 is the main target of 4SC202 in CCA cell apoptosis.

### HDAC3 inhibition induces apoptosis and suppresses cell proliferation in CCA tumor xenografts

**Figure 6:**
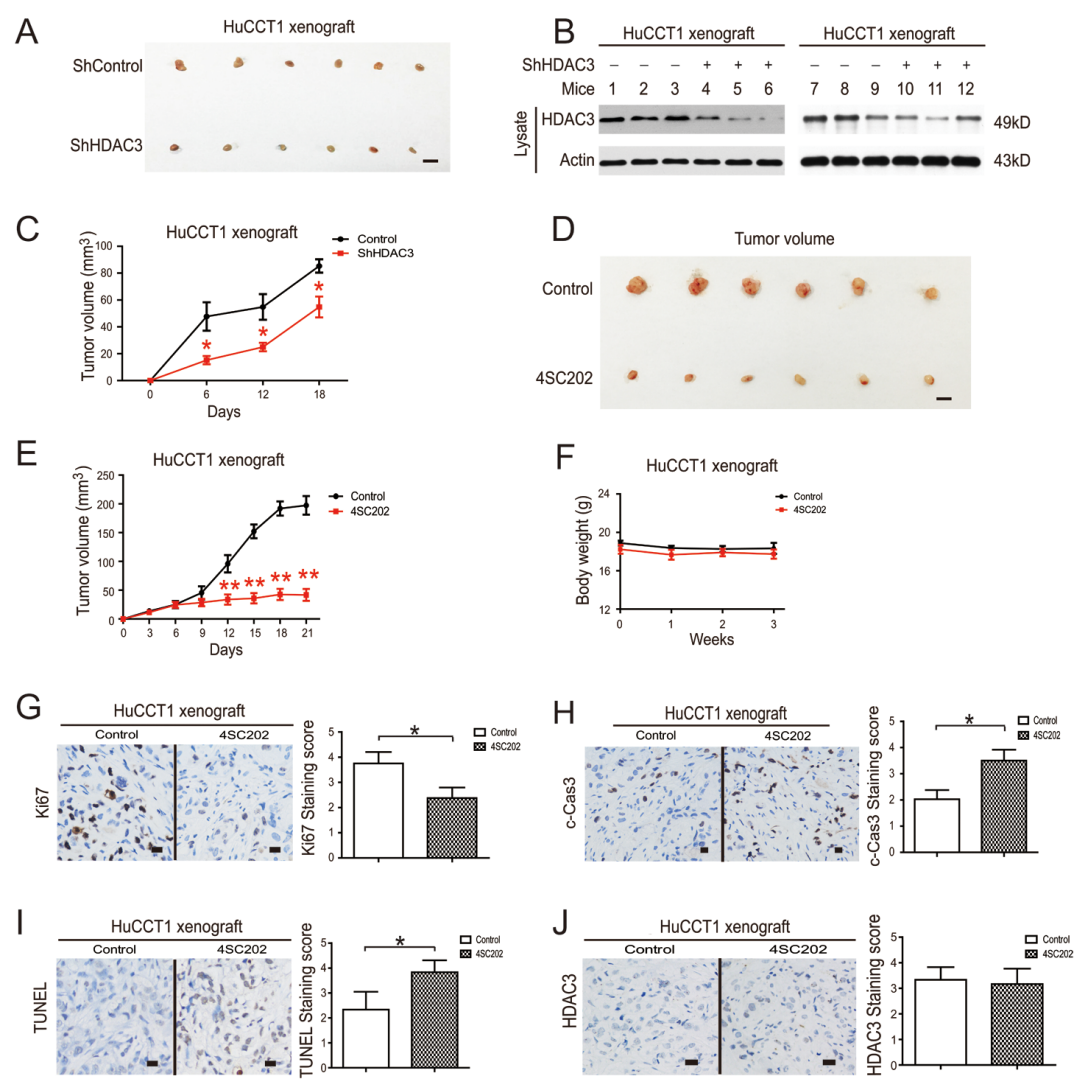
HDAC3 inhibition reduces growth of CCA tumor xenografts **(A)** The tumorigenicity of HDAC3 knockdown cells and their counterparts in nude mice. Tumors were photographed after all animals were sacrificed. Scale bars, 1 cm. **(B)** Xenograft samples of HDAC3 knockdown cells were subjected to western blot for HDAC3. **(C)** The xenograft tumor sizes of HDAC3 knockdown cells and their counterparts. **(D)** Systemic delivery of 4SC202 suppresses CCA cell xenograft tumor growth in nude mice. Tumors were photographed after all animals were sacrificed. Scale bars, 1 cm. **(E)** The xenograft tumor sizes. **(F)** The body weights of tumor-burdened mice. **(G)** Xenograft samples were stained with Ki-67 (left) and staining was quantified (right). **(H)** Xenograft samples were stained with c-Caspase 3 (left) and staining was quantified (right). **(I)** Xenograft samples were stained with TUNEL (left) and staining was quantified (right). **(J)** Xenograft samples were stained with HDAC3 (left) and staining was quantified (right). Data represent the mean ± SEM, n≥3. *p<0.05, **p<0.01, NS not significant.

In order to evaluate the *in vivo* anti-cancer effects of HDAC3 inhibition, we employed a CCA tumor xenograft model and found that HDAC3 knockdown cells also showed a low proliferative capacity and tumorigenicity compared to their counterparts (Figure 6A-6C). 4SC202 administration significantly inhibited tumor growth (Figure 6D & 6E). The body weights of treated mice were used as indicators of health [28]. 4SC202 treatment did not affect mouse body weight, which indicated that the mice did not experience evident toxicity *in vivo* (Figure 6F).

Histological sections of xenograft tumors were analyzed by TUNEL assay, and stained with antibodies against c-caspase 3 and Ki-67, markers of cell apoptosis and proliferation, respectively [28]. Consistent with the *in vitro* results, 4SC202 administration increased TUNEL and c-caspase 3 staining and reduced Ki-67 staining in xenograft tissues, confirming the anti-tumor effect of 4SC202 (Figure 6I). HDAC3 can shuttle in and out of the nucleus, whereas other Class I HDACs are found primarily in the nucleus, as the HDAC3 catalytic domain is positioned much closer to the C-terminus than other Class I HDACs [25]. We found that HDAC3 was mainly localized to the nucleus, but was also observed in the membrane. 4SC202 treatment did not significantly change the location and protein level of HDAC3 in CCA xenograft samples (Figure 6J). These results demonstrate the pro-apoptotic and anti-proliferative effect of HDAC3 inhibition *in vivo*.

## DISCUSSION

Transcriptional repression by class I HDACs (1, 2, 3 and 8) plays a physiological role via the maintenance of cell proliferation as well as the inhibition of specific tumor suppressor genes, thereby resulting in aberrant epigenetics in cancer cells [12, 13, 29, 30]. Since genetic alterations and epigenetic changes are reversible, class I HDACs are suitable for pharmacological intervention [7–11]. Among them, the modifications in HDAC8 expression do not affect cancer cell proliferation[31, 32], and the expression of other class I HDACs in CCA has not been studied. Therefore, we set out to determine the expression of HDAC1, 2, 3 and 8 in CCA tissues. We found that the expression of HDAC1, 2 and 8 showed no significant differences between CCA tissues and adjacent healthy tissues, while HDAC3 expression was increased in CCA tissues and correlated with clinicopathological factors in CCA.

Determining the impact of overexpressed HDAC3 on CCA cells is also of interest, given the important role of epigenetics in carcinogenesis. Acetylation increases p53 protein stability and upon acetylation of p53 at K120, p53 preferentially activates the expression of proapoptotic genes BAX, PUMA, DR5 and NOXA [33]. CCA cells with high HDAC3 expression were found to be resistant to p53-induced apoptosis. The inability of HDAC1 and 2 to activate p53 in these cells indicates that high HDAC3 expression leads to the packaging of the p53 promoter into a highly repressed state. Suppression of HDAC3 in these cells rendered them responsive to p53-induced apoptosis, growth inhibition, and activation of the downstream proapoptotic gene BAX. Increased HDAC3 expression might therefore be an important mechanism for facilitating cancer cell proliferation in CCA. In fact, selective HDAC3 knockdown induced apoptosis and inhibited proliferation of CCA cells. Consistent with these results, HDAC3 overexpression reversed p53-induced apoptosis, while overexpression of HDAC1 and HDAC2 failed to mimic the HDAC3-like effects.

Novel class I HDACs inhibitors (4SC202, BG45, SBHA) were found to be beneficial for cancer cells with good tolerance [5, 21, 22]. Our results suggest that class I HDACs inhibitors negate the growth-promoting function of HDAC3 by suppressing HDAC3 activity and its downstream targets. Because of its lower IC50 to CCA cells *in vitro*, 4SC202 may therefore be an effective agent for preventing CCA carcinogenesis. HDAC 1 and HDAC 2 share 82% identity with each other, while they share 53% and 52% identity with HDAC3, respectively [24–26]. Due to the high level of homology among the class I HDACs, it is easy to comprehend why an HDAC3 selective inhibitor would be difficult to identify. Though 4SC202 can show selectivity for HDAC1, 2 and 3 [34], its inhibitory effects on other HDACs besides HDAC3 cannot be ignored. Therefore, we evaluated the inhibitory effect of 4SC202 on HDACs 2 and 3 by employing mass spectrometry, and confirmed 4SC202 could inhibit HDAC3 activity *in vitro*. At the molecular level, HDAC3 not only partially reversed apoptosis, but also reversed expression of apoptosis-related proteins, whereas HDAC1 and HDAC2 did not have this ability. Consistent with *in vitro* data, 4SC202 significantly inhibited the *in vivo* activity of HDAC3, and induced apoptosis in HuCCT1 xenograft tissues. This data suggests that 4SC202 induces CCA cell apoptosis by mainly targeting HDAC3.

As the catalytic domain of HDAC3 is positioned much closer to the C-terminus than other class I HDACs, the structure of HDAC3 is distinct from other class I HDACs [25]. This may explain why the HDAC3 protein can shuttle in and out of the nucleus, whereas other class I HDACs are found primarily in the nucleus [25]. HDAC3 phosphorylation, which is regulated by the kinase c-Src, casein kinase-2, and by the phosphatase PP4, regulates HDAC3 activity and nuclear localization [35, 36]. We found that HDACs inhibitor treatment did not significantly change the cellular localization or protein levels of HDAC3 in CCA cells and xenograft samples, indicating that the inhibition of HDAC3 activity, instead of its expression and phosphorylation, contributed to the anti-tumor effect of the inhibitor.

In conclusion, this study shows that HDAC3 is a key factor inducing CCA cell proliferation and inhibiting apoptosis, and that increased HDAC3 expression correlates with a poor prognosis in CCA patients. Class I HDAC inhibitors represent a novel treatment approach for CCA. A better understanding of their function in CCA cells is needed to establish their role in the management of CCA.

## MATERIALS AND METHODS

### Ethics

All experiments were approved by the Ethical Committee of Medical Research, Nanjing Drum Tower Hospital, Affiliated Hospital of Nanjing University Medical School. All animal experiments conformed to protocols approved by animal care and use committees at Nanjing University.

### Cell culture and reagents

Six human cholangiocarcinoma (CCA) cell lines HuCCT1, OZ, HuH28, Hccc9810, RBE, and QBC939 were used. HuCCT1, OZ, HuH28, and Hccc9810 cells were obtained from the Japanese Collection of Research Bioresources (JCRB) (Tokyo, Japan). RBE cells were obtained from the Institute of Biochemistry and Cell Biology, Shanghai Institutes for Biological Sciences, Chinese Academy of Sciences (Shanghai, China). QBC939 cells were kindly provided by Professor Shuguang Wang from The Third Military Medical University (Chongqing, China). Cells were maintained in RPMI-1640 medium (Invitrogen, Carlsbad, CA, USA) containing 10% fetal bovine serum (Invitrogen), penicillin (Invitrogen) (100 U/ml) and streptomycin (Invitrogen) (100 U/ml). 4SC202 (Sigma, St Louis, MO, USA) was commercially purchased.

### Immunohistochemistry (IHC)

Tumor specimens were fixed in 4% formalin and embedded in paraffin. Two to five human CCA tumor specimens from one patient were used for the IHC study. The sections were incubated with TUNEL kit buffer (Gugebio, Wuhan, China) or anti-Ki67 antibodies (Santa Cruz, Dallas, TX, USA) and subsequently with DAPI (Gugebio) and the corresponding secondary antibody (Zsbio, Beijing, China). Sections were then treated with immunoperoxidase using the DAB kit (Zsbio) and scored. Staining intensity was graded as follows: absent staining = 0, weak = 1, moderate = 2, and strong = 3. The percentage of staining was graded as follows: 0 (no positive cells), 1 (<25% positive cells), 2 (25% - 50% positive cells), 3 (50% - 75% positive cells), and 4 (>75% positive cells). The score for each tissue was calculated by multiplication and the range of this calculation was therefore 0 – 12 [37].

### Cell transfection

Cells were transfected with Lipofectamine 3000 (Invitrogen) according to the manufacturer’s protocol. The HDAC3 siRNA was commercially purchased from RiboBio (Guangzhou, China), siRNA-HDAC3-1: CCATGACAATGACAAGGAA, siRNA-HDAC3-2: GCATTGATGACCAGAGTTA, siRNA-HDAC3-3: GAATATGTCAAGAGCTTCA. HDAC3 shRNA (h) lentiviral particles were commercially purchased from Santa Cruz Biotechnology. The control vector and HDAC1-3 expression vectors were kindly provided by the Zhao lab of Fudan University (Shanghai, China).

### Western blotting analysis

Cells were lysed with 0.5% NP40 lysis buffer and western blotting was performed according to the standard protocol. Detection was accomplished with the chemiluminescence ECL plus reagent (Thermo, Grand Island, NY, USA) and the chemiluminescence HRP substrate (Millipore, Billerica, MA, USA), and signals were evaluated by a Tanon 5200Multi scanner (Shanghai, China). Primary antibodies were as follows: HDAC1 (Abcam, Cambridge, UK), HDAC2 (Abcam), HDAC3 (Abcam), cleaved caspase-3 (CST, Danvers, MA, USA), cleaved PARP (CST), PARP (CST), GAPDH (Bioworld, St. Louis Park, MN, USA), K9 acetyl-histone H3 (CST), Bax (CST), P53 (Santa Cruz), PUMA (CST), and HA (CST).

### Cell viability and clonogenic assay

Cell viability was assessed by the CCK-8 colorimetric assay in 96-well plates (2×10^3^ cells/well) (Dijindo, Minato-ku, Tokyo, Japan). The absorbance at 450 nm was recorded using a micro-plate reader. For the clonogenic assay, cells were seeded into 6-well plates at a density of 500 cells/well. Following culture for 10 days, individual colonies were counted after crystal violet staining.

### Apoptosis assay

Cells were analyzed for apoptosis with the AnnexinV-FITC/PI Apoptosis Detection Kit (BD, Franklin Lakes, NJ, USA) following the manufacturer's instructions.

### HDAC deacetylation assay

Cells were lysed in NP-40 buffer containing 50 mM Tris-HCl (pH 7.5) (Sigma, St Louis, MO, USA), 150 mM NaCl (Sangon, Shanghai, China), 0.5% Nonidet P-40 (Sigma), 1 μg/ml aprotinin (Sigma), 1 μg/ml leupeptin (Sigma), 1 μg/ml pepstatin (Sigma), 1 mM Na_3_VO_4_ (Sigma) and 1 mM PMSF (Sigma). For immunoprecipitation, 500 μl of cell lysate was incubated with HA antibody (provided by the Zhao lab of Fudan University) for three hours at 4°C with rotation. Then, 30 μl of Protein A Agarose (Millipore) was added for 12 hours at 4°C with rotation, and the beads were washed three times with lysis buffer before proteins were dissolved in loading buffer. Deacetylation assays were carried out in the presence of 5 μg enzyme and 0.3 μg peptide in 30 μl reaction buffer [30 mM HEPES (Sigma), 0.6 mM MgCl_2_ (Sangon), 1 mM DTT (Sigma), 1 mM NAD^+^ (Sigma), 10 mM PMSF (Sigma)]. The deacetylation reaction was incubated for 3 – 5 hours at 37°C before the mixture was desalted by passing it through a C18 ZipTip (Millipore). The desalted samples were analyzed using a MALDI-TOF/TOF mass spectrometer (Applied Biosystems, Grand Island, NY, USA). The acetylated peptide used in the assay was NLASVEELK^Ac^EIDVEVRK (Glssale, Shanghai, China).

### Cholangiocarcinoma cancer xenograft model

Nude mice were purchased from the Department of Laboratory Animal Science, Nanjing Drum Tower Hospital. HuCCT1 cells (5×10^6^) in FBS-free RPMI-1640 were subcutaneously injected into the flanks of mice. When tumors were measurable, mice were treated with intraperitoneal injection (IP) of vehicle control or 4SC202 (50 mg/kg) in 200 μl volume twice a week for three weeks. HDAC3 knockdown HuCCT1 cells and their counterparts were injected into the flanks of the same mice. Tumor volume was calculated using the formula, length (L) x width (W) x height (H) x 0.5236. The Animal Welfare Committee of Nanjing Drum Tower Hospital approved all procedures involving animals.

### Statistics

Data are expressed as means ± standard error of the mean (SE). The data were analyzed through one-way ANOVAs followed by post hoc Duncan tests (SPSS 17.0). P<0.05 was considered significant.
